# Polyamine-metabolizing enzymes are activated to promote the proper assembly of rice stripe mosaic virus in insect vectors

**DOI:** 10.1007/s44154-021-00032-z

**Published:** 2022-04-15

**Authors:** Dongsheng Jia, Huan Liu, Jian Zhang, Wenqiang Wan, Zongwen Wang, Xiaofeng Zhang, Qian Chen, Taiyun Wei

**Affiliations:** Fujian Province Key Laboratory of Plant Virology, State Key Laboratory of Ecological Pest Control for Fujian and Taiwan Crops, Fujian Agriculture and Forestry University, Fuzhou, Fujian People’s Republic of China

**Keywords:** Rice stripe mosaic virus, Rhabdovirus, Insect vector, Polyamines, OAZ1, ODC1, Viral assembly

## Abstract

Both viruses and host cells compete for intracellular polyamines for efficient propagation. Currently, how the key polyamine-metabolizing enzymes, including ornithine decarboxylase 1 (ODC1) and its antizyme 1 (OAZ1), are activated to co-ordinate viral propagation and polyamine biosynthesis remains unknown. Here, we report that the matrix protein of rice stripe mosaic virus (RSMV), a cytorhabdovirus, directly hijacks OAZ1 to ensure the proper assembly of rigid bacilliform non-enveloped virions in leafhopper vector. Viral matrix protein effectively competes with ODC1 to bind to OAZ1, and thus, the ability of OAZ1 to target and mediate the degradation of ODC1 is significantly inhibited during viral propagation, which finally promotes polyamines production. Thus, OAZ1 and ODC1 are activated to synergistically promote viral persistent propagation and polyamine biosynthesis in viruliferous vectors. Our data suggest that it is a novel mechanism for rhabdovirus to exploit OAZ1 for facilitating viral assembly.

**Supplementary Information:**

The online version contains supplementary material available at 10.1007/s44154-021-00032-z.

## Introduction

Viruses utilize and alter cellular metabolic pathways in host cells for their successful infection. Polyamines, consisting of putrescine (PUT), spermidine (SPD) and spermine (SPM), are small, positively charged molecules that are essential for normal cell growth and viability in eukaryotic cells (Bae et al., 2018; Handa et al., 2018). Viruses also rely on intracellular polyamines for infection (Mounce et al., 2017; Firpo & Mounce, 2020). Thus, viruses and host cells compete for intracellular polyamines as they are critical resources for both. Polyamine homeostasis is tightly regulated by the enzymes in their biosynthetic pathway, including ornithine decarboxylase (ODC1), ornithine decarboxylase antienzyme (OAZ1), spermidine/spermine-N1-acetyl transferase (SAT) and spermine oxidase (SMO) (Mounce et al., 2017; Kahana, 2018). OAZ1 is a central player in the regulatory circuit that controls cellular levels of polyamines by regulating ODC1 degradation (Kahana, 2018; Wu et al., 2015). ODC1, the first rate-limiting enzyme in polyamine biosynthesis, regulates the conversion of ornithine to PUT (Wu et al., 2015; Kahana, 2018). OAZ1 binding promotes the dissociation of ODC1 homodimers to form OAZ1-ODC1 heterodimers, abolishing ODC1 enzyme activity and targeting ODC1 for degradation by the 26S proteasome (Wu et al., 2015; Kahana, 2018). The C-terminal region of OAZ1 is essential and fully functional for the binding, inhibition, and degradation of ODC1 (Hsieh et al., 2011; Kahana, 2018). The OAZ1 mRNA transcript contains two overlapping open reading frames (ORFs). The rising intracellular polyamine concentrations would induce a + 1 ribosomal frameshift of the OAZ1 mRNA, which produces a longer functional OAZ1 protein (Rom & Kahana, 1994; Matsufuji et al., 1995). Hepatitis C virus and some herpesviruses, including Epstein-Barr virus, herpes simplex virus, bovine herpesvirus and human cytomegalovirus, have been shown to manipulate polyamine levels by inducing the expression of these enzymes (Firpo & Mounce, 2020). Currently, how the polyamine-metabolizing enzymes are activated to co-ordinate viral propagation and polyamine biosynthesis remains unknown.

Rhabdoviruses form a large family whose collective host range includes vertebrates, invertebrates, and plants, and are of considerable socioeconomic and agricultural importance (Waiker et al., 2018). For example, rabies virus (RABV) causes lethal encephalitis that results in approximately 50,000 human deaths per year (Katz et al., 2017). Plant rhabdoviruses that are transmitted by insect vectors in a persistent-propagative manner cause substantial agricultural losses (Di et al., 2014; Yang et al., 2017; Liu et al., 2018; Chen et al., 2019; Fang et al., 2019). According the different replication and assembly place, plant rhabdoviruses with unsegmented genomes have been taxonomically classified into the genera *Cytorhabdovirus*, and *Nucleorhabdovirus* (Whitfield et al., 2018). Rhabdovirus virions are typically enveloped with bullet-shaped or bacilliform morphology with negative-sense (−) single-stranded RNA genomes encoding five structural proteins (Walker et al., 2018). During viral assembly, nucleoprotein (N), polymerase (L), phosphoprotein (P) and RNA genome form the ribonucleoprotein (RNP) core (Jayakar et al., 2004; Ivanov et al., 2011). The replication and assembly of rhabdoviruses take place in viral inclusions called viroplasms in the nuclei or cytoplasms of infected cells (Jackson et al., 2005; Dietzgen et al., 2020). Viroplasm matrix induced by cytorhabdoviruses is basically composed of P, and then N is recruited into the viroplasm by interaction with P (Fang et al., 2019; Zhang et al., 2021). Generally, the RNP cores are constructed inside the viroplasms, whereas matrix proteins (M) condense the RNP cores to assemble into non-enveloped virions at the periphery of viroplasms (Jayakar et al., 2004; Ivanov et al., 2011). During the maturation of rhabdoviruses, the condensed non-enveloped virions ultimately bud through host membranes to acquire the lipid envelope and glycoprotein (G) to assemble mature enveloped virions (Jayakar et al., 2004; Ivanov et al., 2011). Previous studies have shown that polyamine depletion by difluormethylornithine (DFMO), a specific nontoxic inhibitor of ODC1, could inhibit the infection of mammalian cells by vertebrate rhabdoviruses, including RABV and vesicular stomatitis virus (VSV) (Mounce et al., 2016). However, how polyamine-metabolizing enzymes are activated to synergistically promote rhabdovirus propagation and polyamine biosynthesis has yet to be elucidated.

Recently, we demonstrated that the nucleorhabdovirus rice yellow stunt virus (RYSV) could activate PUT biosynthesis in leafhopper vector to promote viral propagation by inhibiting OAZ1 expression (Zhang et al., 2021). We further showed that the infection of RYSV in leafhopper vector upregulates ODC1 expression but inhibits OAZ1 expression, which would facilitate the conversion of ornithine to PUT (Zhang et al., 2021). However, in this study, we find that the infection of the cytorhabdovirus rice stripe mosaic virus (RSMV) in leafhopper vector could significantly upregulates both OAZ1 and ODC1 expression, which also facilitates the conversion of ornithine to PUT expression. RSMV is transmitted by its rice green leafhopper vector *Recilia dorsalis* (Hemiptera: Cicadellidae) in a persistent-propagative manner and has recently spread rapidly throughout southern China (Yang et al., 2018; Chen et al., 2019). The replication and assembly of rigid bacilliform non-enveloped virions of RSMV occur at the periphery of viroplasm in the cytoplasm of insect vectors (Zhao et al., 2019; Wang et al., 2020). Here, we report that OAZ1 is activated to promote the proper assembly of RSMV in insect vectors. We further determine how RSMV, OAZ1 and ODC1 have formed a delicate regulatory circuit to co-ordinate polyamine biosynthesis and viral persistent propagation in insect vectors.

## Results

### RSMV M competes with ODC1 to bind OAZ1 in vitro

**Fig. 1:**
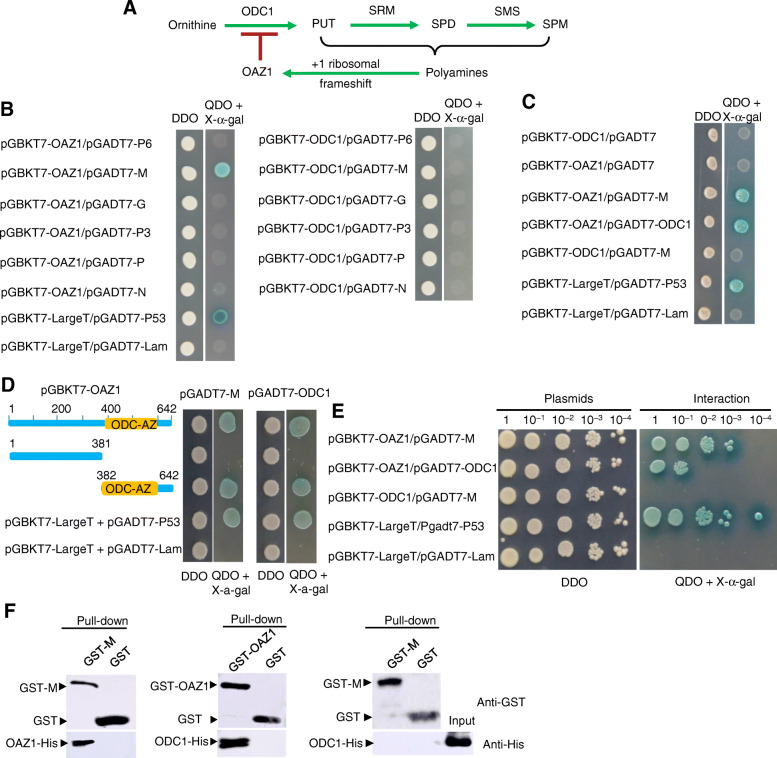
RSMV M competes with ODC1 to bind OAZ1. **A** The proposed model for the polyamine synthesis pathway in *R. dorsalis*. **B** Interactions among M, N, P, G, P3 or P6 of RSMV and ODC1 or OAZ1 of *R. dorsalis* were detected by yeast two-hybrid assay. Transformants on plates of DDO and QDO + X-α-gal were shown. **C** Interactions among RSMV M, OAZ1 and ODC1 were detected by yeast two-hybrid assay. Transformants on plates of DDO and QDO + X-α-gal were shown. **D** The ODC-AZ domain of OAZ1 interacted with RSMV M and ODC1, as detected by yeast two-hybrid assay. **E** The competitive interactions among RSMV M, OAZ1 and ODC1 were detected by a serially-diluted yeast two-hybrid assay. The yeast strain AH109 was co-transformed with pGBKT7-OAZ1/pGADT7, pGBKT7-ODC1/pGADT7, pGBKT7-OAZ1/pGADT7-M, pGBKT7-OAZ1/pGADT7-M or pGBKT7-ODC1/pGADT7-M. pGBKT7-LargeT/pGADT7-P53 was used as the positive control; pGBKT7-LargeT/pGADT7-Lam served as the negative control. After being diluted to 10^− 1^ to 10^− 4^, the yeast cells were plated onto DDO or QDO + X-α-gal plates. **F** GST pull-down assay was used to detect interactions among M, ODC1 and OAZ1. OAZ1 or RSMV M fused with GST served as the bait, and single GST served as the control. OAZ1 or ODC1 fused with His served as the prey. Input and pull-down samples were probed with antibodies against GST or His for western blot assay

Firstly, we investigated the interaction of RSMV-encoded proteins with the two key polyamine-metabolizing enzymes OAZ1 and ODC1 from *R. dorsalis*. We cloned the full-length of OAZ1 and ODC1 genes based on the bioinformatics analysis of leafhopper RNA-seq result. The OAZ1 gene of *R. dorsalis* contained 642 bp and encoded two overlapping ORFs with a conserved + 1 ribosomal frameshift positioned at the + 160 T base (Fig. 1A and S1, GenBank accession number MN931685). The second ORF of OAZ1 encoded a 213 amino acid protein with the characteristic ODC1 binding domain at the C-terminus of OAZ1 (ODC-AZ domain) (Fig. S1). In this study, we created an OAZ1 frameshift mutant at the + 160 T base to encode the full length of OAZ1 protein. The ODC1 gene of *R. dorsalis* contained 1311 bp and encoded a 437 amino acid protein (Fig. S2, GenBank accession number MN854702).

We then investigated whether OAZ1 or ODC1 of *R. dorsalis* could interact with any of the RSMV-encoded proteins (N, P, P3, M, P6 and G). Yeast two-hybrid assay showed that, of the six RSMV-encoded proteins, only M specifically interacted with OAZ1 but not with ODC1 (Fig. 1B, C). Furthermore, both RSMV M and ODC1 interacted with the ODC-AZ domain of OAZ1 (Fig. 1D). It appeared that the interaction between OAZ1 and RSMV M was stronger than the interaction between OAZ1 and ODC1 (Fig. 1E). These interactions were independently verified by glutathione S-transferase (GST) pull-down assay (Fig. 1F). Taken together, these results suggested that RSMV M competed with ODC1 to bind OAZ1, thereby releasing OAZ1 from its inhibiting the function of ODC1 during viral infection in insect vectors.

### OAZ1 is recruited by RSMV M to support the assembly of rigid bacilliform non-enveloped virions in vivo

**Fig. 2:**
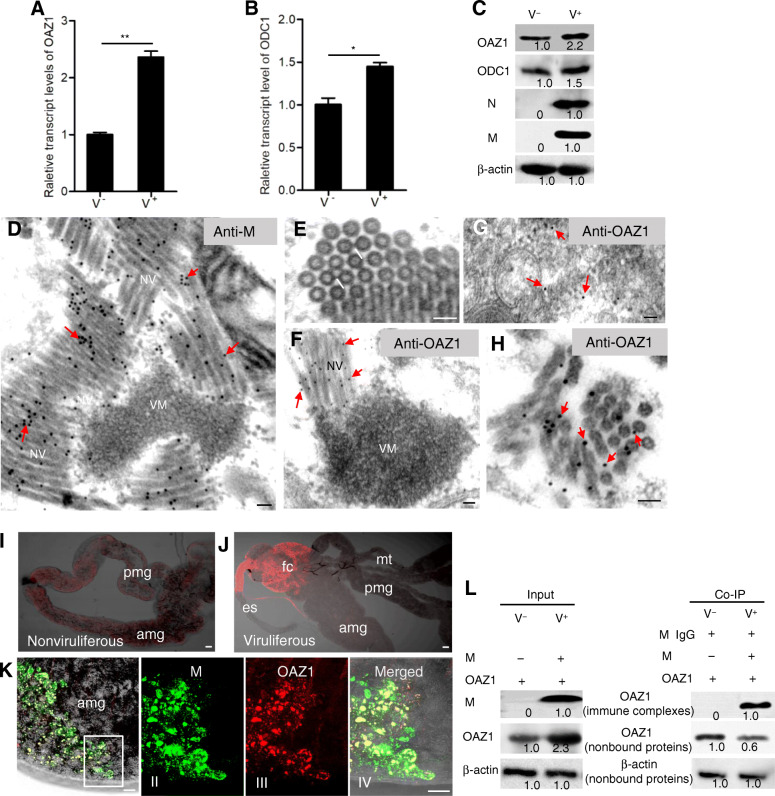
The association of OAZ1 with the rigid bacilliform non-enveloped virions of RSMV in *R. dorsalis*. **A, B** The transcript levels of OAZ1 (**A**) and ODC1 (**B**) in viruliferous and nonviruliferous insects detected by RT-qPCR assay. Data are presented as means (± standard deviation) from three independent experiments (Student’s *t*-test, two tailed). *, *P* < 0.05. **, *P* < 0.01. **C** The protein levels of OAZ1, ODC1, M or N in viruliferous and nonviruliferous insects, as detected by western blot assay by using OAZ1-, ODC1-, M- or N-specific IgGs. Insect β-actin was used as an internal control. **D** Immunogold labeling of RSMV M on the rigid bacilliform non-enveloped virions at the periphery of viroplasms in virus-infected intestines. Insect intestines were immunolabeled with M-specific antibodies and goat antibodies against rabbit IgG that had been conjugated with 15-nm-diameter gold particles as secondary antibodies. **E** The cross section of rigid bacilliform particles, which were connected by the filaments (lines) within the paracrystalline array. **F** Immunogold labeling of OAZ1 on the rigid bacilliform non-enveloped virions at the periphery of viroplasm matrix in virus-infected intestines. **G** The diffuse distribution of OAZ1 in nonviruliferous insect intestine. **H** Immunogold labeling of OAZ1 on the margin of particles or at the space between two adjacent particles. Insect intestines were immunolabeled with M- (**D**) or OAZ1-specific (**F-H**) antibodies and goat antibodies against rabbit IgG that had been conjugated with 15-nm-diameter gold particles as secondary antibodies. Red arrows mark gold particles. Bars, 50 nm. **I, J** The different distributions of OAZ1-rhodamine (red) in the whole intestines of nonviruliferous (**I**) or viruliferous (**J**) insects at 4 days padp. **K** Immunofluorescence microscopy showing OAZ1 was always accompanied with RSMV M in the intestines of viruliferous insects. RSMV-infected insect intestines were immunolabeled with M-FITC (green) and OAZ1-rhodamine (red) at 4 days padp. Panels II-V are the enlargements of the boxed areas in panel I. Bars, 30 μm. **L** The lysates from viruliferous or nonviruliferous insects were immunoprecipitated with M antibodies, and the resultant immune complexes and nonbound proteins were detected by OAZ1- or β-actin- specific antibodies. Meanwhile, the actin, M and OAZ1 proteins in the input lysate were detected by actin-, M- and OAZ1-specific antibodies, respectively. V^−^, nonviruliferous; V^+^, viruliferous; es, esophagus; fc, filter chamber; amg, anterior midgut; pmg, posterior midgut; mt, midgut; VM, viroplasm matrix; NV, non-enveloped virions

RT-qPCR and western blot assays showed that the mRNA and protein levels of OAZ1 and ODC1 were both upregulated during viral infection in insect vectors (Fig. 2A, B, C). It appeared that the increased expression of OAZ1 did not mediate the degradation of ODC1 in viruliferous *R. dorsalis*. We thus investigated how the direct interaction of RSMV M and OAZ1 facilitated the assembly of non-enveloped virions in the intestines of *R. dorsalis*. Rhabdovirus RNP cores are constructed inside the viroplasm while M proteins are condensed to the RNP cores to assemble rigid non-enveloped virions at the periphery of the viroplasm (Jackson et al., 2005; Ammar et al., 2009). Immunoelectron microscopy indicated that RSMV N was localized to the viroplasm matrix (Fig. S3A); however, RSMV M was localized to the rigid bacilliform non-enveloped virions of 45 to 65 nm in width and 180 to 320 nm in length at the periphery of viroplasm matrix (Fig. 2D, S3B). These non-enveloped virions were aligned in parallel and tightly packed to form paracrystalline arrays (Fig. 2D, S3B). In cross section, individual rigid bacilliform particles were connected by the thin filaments within the paracrystalline array (Fig. 2E). Electron microscopy showed that the rigid bacilliform non-enveloped virions that aligned in parallel and tightly packed were accumulated adjacent to the endoplasmic reticulum (ER) membrane (Fig. S3C). Importantly, we observed that one end of these rigid bacilliform particles attached to the cytoplasmic face of the ER membrane, which finally mediated the budding of non-enveloped particles into the ER cisternae to assemble the intact enveloped virions of approximately 90 nm in width and 180 to 320 nm in length (Fig. S3D). RSMV G antibodies specifically reacted with these enveloped virions within the ER cisternae (Fig. S3D).

Immunoelectron microscopy confirmed that OAZ1 antibody specifically reacted with the rigid bacilliform non-enveloped but not with the matrix of viroplasm in virus-infected regions (Fig. 2F). In contrast, OAZ1 was only sparsely present in virus-free regions (Fig. 2G). In cross section, OAZ1 also was localized at the space between adjacent bacilliform particles within the paracrystalline array and present on the virions (Fig. 2H). Immunofluorescence microscopy confirmed that OAZ1 was diffusely distributed in the cytoplasm of intestinal epithelial cells of nonviruliferous insects (Fig. 2I). However, OAZ1 was always accompanied by RSMV M in viruliferous insect intestines (Fig. 2J, K). Furthermore, immunofluorescence and immunoelectron microscopy showed that ODC1 was always sparsely distributed in the cytoplasm, but not associated with non-enveloped virions (Fig. S4). It was clear that the elevated contents of OAZ1 were mainly restricted to virus-infected regions, possibly facilitating the assembly of rigid bacilliform non-enveloped virions through the direct interaction of OAZ1 and RSMV M. To confirm this, the intestines from nonviruliferous or viruliferous insects were subjected to immunoprecipitation with RSMV M antibody. This resulted in the co-immunoprecipitation of large amounts of OAZ1 by M antibody from viruliferous insects, but not from nonviruliferous insects (Fig. 2L). More importantly, the OAZ1 contents in the nonbound proteins from viruliferous insects were significantly lower than that from nonviruliferous controls (Fig. 2L). These in-vivo experiments further suggested that OAZ1 contents in virus-infected regions were mostly recruited by RSMV M, and thus the accumulation level of M-nonbound OAZ1 in virus-infected regions was significantly decreased, confirming that RSMV M could effectively compete with ODC1 to bind OAZ1.

### RSMV infection increases polyamines production to promote viral propagation and transmission by *R. dorsalis*

**Fig. 3:**
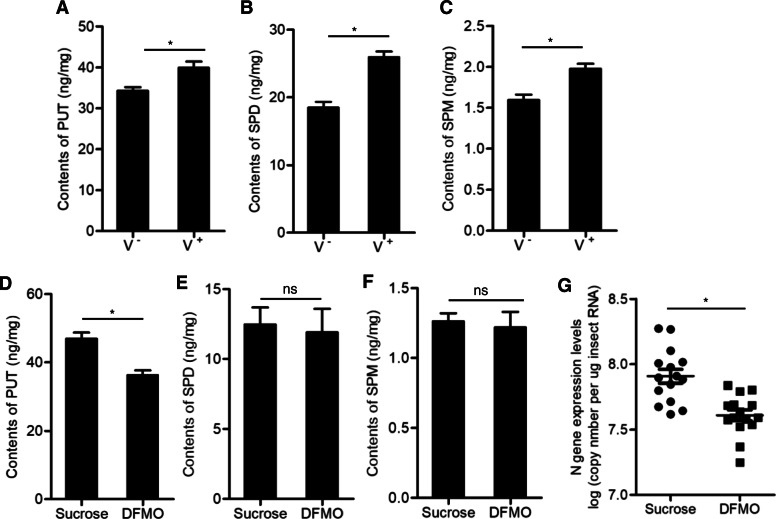
RSMV infection increased polyamines production in *R. dorsalis*. **A-C** The contents of PUT (**A**), SPD (**B**) and SPM (**C**) in viruliferous or nonviruliferous insects, as detected by UPLC-MS/MS assay. **D-F** The contents of PUT (**D**), SPD (**E**) and SPM (**F**) in viruliferous insects treated with DFMO or sucrose control, as detected by UPLC-MS/MS assay. Data in **A**-**F** are presented as means (± standard deviation) from three independent experiments (Student’s *t*-test, two tailed). *, *P* < 0.05. ns, non-significant. **G** The expression levels of RSMV N in viruliferous insects treated with DFMO or sucrose control. The N gene copy number in individual insects was detected by RT-qPCR assay. Each horizontal line represents the mean gene copy number of each data set. The significance of difference was tested using Tukey’s HSD test. *, *P* < 0.05

In insects, the polyamine PUT is biosynthesized via the activity of ODC1, which is the first enzyme in polyamines biosynthesis and is regulated by OAZ1 (Fig. 1A). To determine whether RSMV infection increased the abundance of polyamines production, we compared the concentrations of three polyamines including PUT, SPD and SPM in virus-infected *R. dorsalis* vectors by using ultra-performance liquid chromatography-mass spectrometry (UPLC-MS/MS) assay. The contents of all three polyamines were increased during viral infection in insect vectors (Fig. 3A-C). DFMO, a suicide inhibitor of ODC1 (Mounce et al., 2016), was used to inhibit polyamine biosynthesis during viral infection in insect vectors. Treatment with 0.5 mM DFMO resulted in the significant decreases of intracellular PUT production (Fig. 3D-F), confirming the role of ODC1 in regulating the conversion of ornithine to PUT. Furthermore, DFMO treatment led to a significant inhibition of viral accumulation (Fig. 3G), finally decreasing viral transmission rates by *R. dorsalis* to rice plants (Table S1). Thus, the upregulation of ODC1 expression level during RSMV infection increased polyamines production in *R. dorsalis* (Fig. 2, 3B, A-C).

### The knockdown of OAZ1 expression inhibits efficient viral propagation in insect vectors

**Fig. 4:**
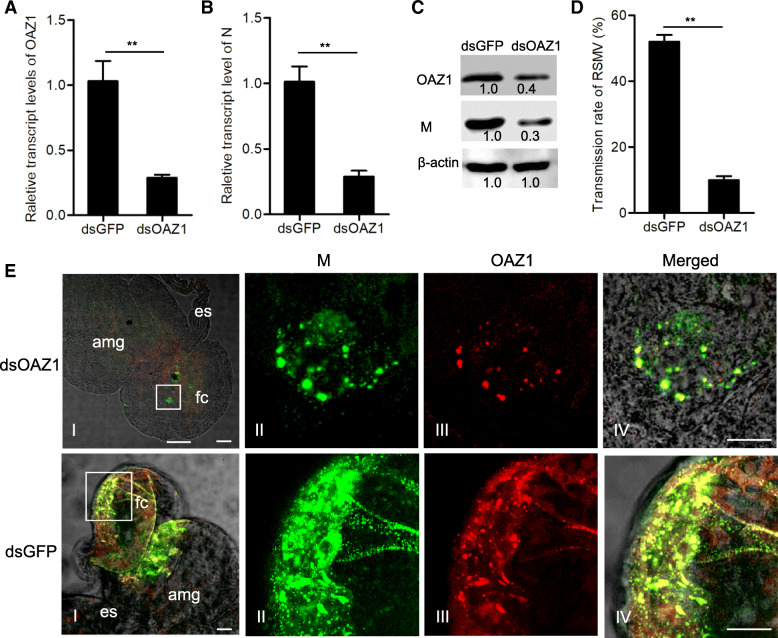
Knockdown of OAZ1 expression inhibited viral infection and transmission by *R. dorsalis*. **A, B** The transcript levels of OAZ1 (**A**) and M (**B**) in viruliferous insects after treatment of dsGFP or dsOAZ1 were measured by RT-qPCR assay. **C** The protein levels of OAZ1 and M in viruliferous insects after treatment of dsGFP or dsOAZ1 were detected by western blot assay using OAZ1- and M-specific IgGs. Insect β-actin was detected with β-actin-specific IgG as an internal control. **D** Transmission rates of RSMV by dsGFP- or dsOAZ1-treated *R. dorsalis* individuals. **E** The intestines of viruliferous insects after treatment of dsOAZ1 or dsGFP were immunolabeled with M-FITC (green) and OAZ1-rhodamine (red) at 4 days padp. es, esophagus; fc, filter chamber; amg, anterior midgut. Panels II-IV are the enlargements of the boxed areas in panels I. Bars, 30 μm. Data in **A**, **B** and **D** are presented as means (± standard deviation) from three independent experiments (Student’s *t*-test, two tailed). **, *P* < 0.01

We then microinjected *R. dorsalis* with the in vitro synthesized dsRNAs targeting the OAZ1 gene (dsOAZ1) to confirm whether OAZ1 could facilitate viral propagation. The mortality rates of *R. dorsalis* were not significantly changed after the microinjection of dsOAZ1 (Fig. S5). As expected, the treatment of dsOAZ1 significantly knocked down the in vivo expression of OAZ1 (Fig. 4A, C). In addition, RT-qPCR and western blot assays demonstrated that the reduced expression of OAZ1 caused a significant decrease of viral accumulation in insect vectors (Fig. 4B, C), finally decreasing viral transmission rate by *R. dorsalis* into rice plants (Fig. 4D and Table S1). Immunofluorescence microscopy confirmed that the reduced expression of OAZ1 significantly inhibited viral infection in the intestinal epithelium of viruliferous insects (Fig. 4E). Taken together, these data suggested that OAZ1 was essential for efficient viral propagation in insect vectors.

### OAZ1 is essential for the formation of bacilliform structures of RSMV M in vitro

**Fig. 5:**
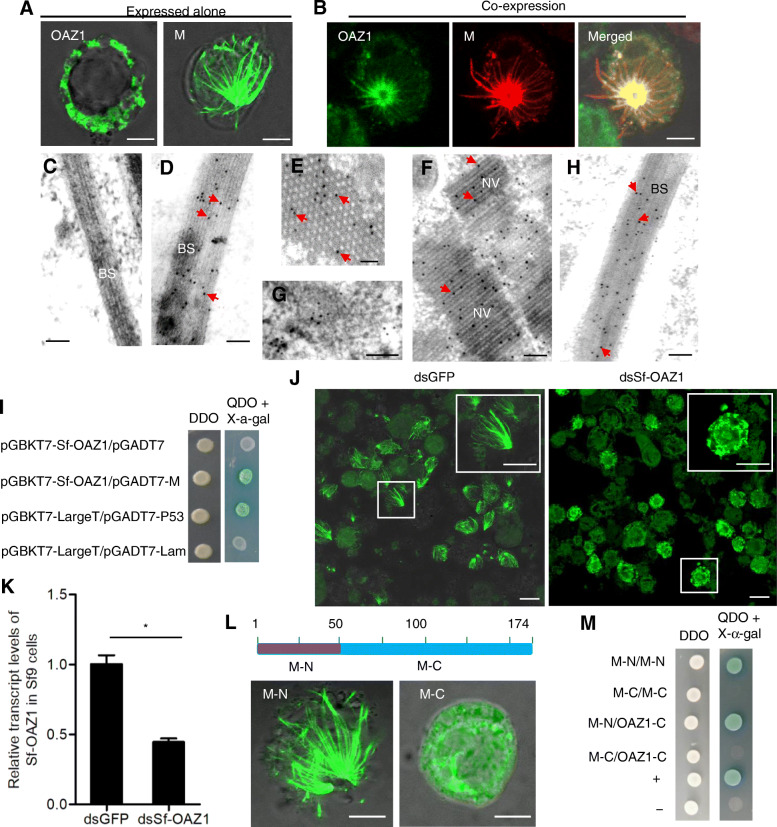
OAZ1 is involved in the formation of bacilliform structures of RSMV M in Sf9 cells. **A, B** The interactions between RSMV M and OAZ1 in Sf9 cells. M and OAZ1 were expressed alone (**A**) or co-expressed (**B**). Sf9 cells were fixed at 48 hpi and immunolabeled with OAZ1-FITC (green) or M-specific IgG conjugated to rhodamine (M-rhodamine) (red). Bars, 5 μm. **C-H** Immunoelectron microscopy showing that RSMV M formed the bacilliform structures (**D, E**) in Sf9 cells, which are similar in appearance to the rigid bacilliform non-enveloped virions (**F**) in *R. dorsalis*. The longitudinal and cross sections of the bacilliform structures of M (**C, E**) and a longitudinal section of non-enveloped virions (**F**) were shown. Bars, 100 nm. **G, H** Immunogold labelling of OAZ1 in Sf9 cells expressing OAZ1 alone (**G**) or co-expressing OAZ1 and RSMV M (**H**). Sf9 cells were fixed at 48 hpi and immunolabeled with M- or OAZ1-specific IgGs, and goat antibodies against rabbit IgG that had been conjugated with 15-nm-diameter gold particles (arrows) as secondary antibodies. NV, non-enveloped virions. BS, bacilliform structure. Red arrows, gold particles. Bars, 100 nm. **I** Yeast two-hybrid assay showing OAZ1 from Sf9 cells (Sf-OAZ1) interacted with RSMV M. Transformants on plates of DDO and QDO + X-α-gal are shown. **J** The dsGFP- or dsSf-OAZ1-treated Sf9 cells infected with recombinant baculovirus expressing M were fixed at 48 hpi and immunolabeled with M-FITC (green). Inserts are the enlargements of the boxed areas in each panel. Bars, 5 μm. **K** The transcript levels of Sf-OAZ1 in Sf9 cells infected with recombinant baculovirus expressing RSMV M after treatment of dsGFP or dsSf-OAZ1 were detected by RT-qPCR assay. Means (± standard deviation) from three biological replicates are shown. *, *P* < 0.05. **L** Immunofluorescence assay showing that the N-terminal region (1–50 aa, M-N) but not the C-terminal region (51–174 aa, M-C) of RSMV M formed the bacilliform structures in Sf9 cells. Sf9 cells expressing the N- or C-terminal regions of RSMV M were fixed at 48 hpi and immunolabeled with M-FITC (green). Bars, 5 μm. **M** Yeast two-hybrid assay showing the N-terminal region of RSMV M interacted with itself and the ODC-AZ domain of OAZ1. The C-terminal region of RSMV M did not interact with itself or the ODC-AZ domain of OAZ1. Transformants on plates of DDO and QDO + X-α-gal are shown

We then used an in vitro baculovirus expression system to investigate the relationship of RSMV M and OAZ1. When expressed alone, RSMV M formed the filament-like structures, while OAZ1 was diffusely distributed in the cytoplasm of Sf9 cells (Fig. 5A). The co-expression led to the redistribution of OAZ1 into the filament-like structures of M (Fig. 5B). Electron microscopy confirmed that RSMV M formed the bacilliform structures of 30–45 nm in width in the cytoplasm of Sf9 cells (Fig. 5C-E). These bacilliform structures were aligned in parallel and tightly packed, in an appearance similar to the rigid bacilliform non-enveloped virions (Fig. 5F). Thus, RSMV M had an intrinsic ability to form the bacilliform structures for the packaging of RNPs. Immunoelectron microscopy confirmed that OAZ1 antibody specifically recognized the bacilliform structures (Fig. 5G, H).

We then investigated whether the knockdown of the expression of OAZ1 in Sf9 cells (Sf-OAZ1) by RNAi would affect the formation of bacilliform structures of RSMV M in vitro. Yeast two-hybrid assay showed that the Sf-OAZ1 interacted with RSMV M (Fig. 5I). The synthesized dsRNAs targeting the segments of Sf-OAZ1 (dsSf-OAZ1) were prepared and transfected into Sf9 cells that had been infected with recombinant baculovirus expressing RSMV M. The reduced expression of Sf-OAZ1 significantly inhibited the ability of RSMV M to form the bacilliform structures (Fig. 5J, K). Thus, OAZ1 was involved in the biogenesis of the bacilliform structures formed by RSMV M, even in the absence of other viral proteins and virus propagation.

Distantly-related rhabdovirus M proteins have similar coiled helical structures that confer the rigidity of the non-enveloped virions (Graham et al., 2008). Secondary structure prediction showed that RSMV M contained two conserved α-helices in the N-terminal region (1–50 aa) and an α-helix in the C-terminal region (51–174 aa) (Fig. S6). Expression of the N-terminus of RSMV M led to the formation of the bacilliform structures, but the expressed C-terminus of RSMV M was diffusely distributed in the cytoplasm (Fig. 5L). Yeast two-hybrid assay showed that the N-terminus but not the C-terminus of RSMV M directly interacted with itself (Fig. 5M). In addition, the ODC-AZ domain of OAZ1 interacted with the N-terminus but not with the C-terminus of RSMV M (Fig. 5M). Taken together, these results demonstrated that OAZ1 facilitated the ability of the N-terminus of RSMV M to form the rigid bacilliform structures.

## Discussion

**Fig. 6:**
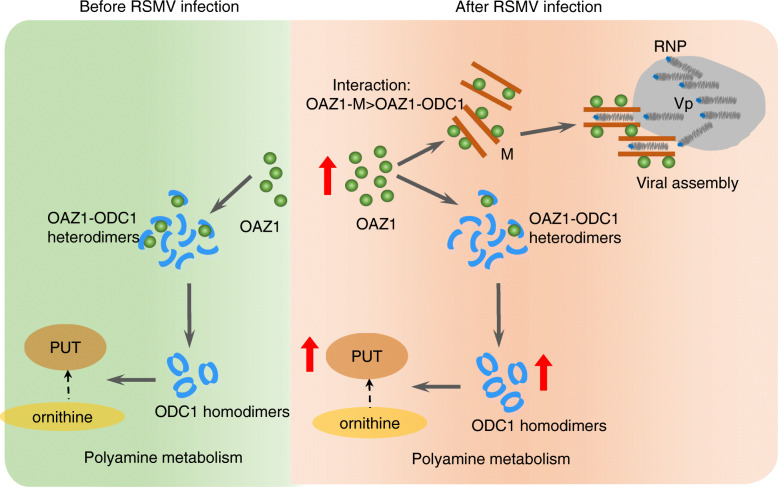
The proposed model for the activation of OAZ1 by RSMV infection to promote polyamines production and viral assembly in insect vectors. In virus-infected cells, the elevated OAZ1 contents are mostly recruited by RSMV M to promote viral assembly at the periphery of viroplasm. This process decreases the ability for OAZ1 to target and mediate the degradation of ODC1, finally leading to the increase of ODC1 contents to promote the conversion of ornithine to Put. RNP, ribonucleoprotein; M, matrix protein; Vp, viroplasm; PUT, putrescine

From the 1950s to the present day, a broad base of literatures surrounding polyamines and viruses suggests that these small molecules have a big impact on virus infections. However, the precise roles of the polyamine-metabolizing enzymes in viral propagation remain unclear. OAZ1, a small polyamine-induced protein, is a central player in an autoregulatory loop that modulates cellular polyamine levels (Kahana, 2018). During RSMV propagation in insect vectors, RSMV M recognizes and condenses RNP cores to assemble abundant rigid bacilliform non-enveloped virions at the periphery of viroplasm. We find that the intracellular accumulation level of OAZ1 is significantly increased to support viral assembly. Both RSMV M and ODC1 compete to directly bind to the ODC-AZ domain of OAZ1; however, the binding affinity of RSMV M with OAZ1 is stronger than that of ODC1 with OAZ1. In virus-infected regions, OAZ1 contents are mostly recruited by RSMV M; accordingly, M-nonbound OAZ1 contents for targeting ODC1 are significantly decreased. Thus, during viral infection, the ability for OAZ1 to mediate the degradation of ODC1 is significantly inhibited, finally leading to the increase of ODC1 in the polyamine biosynthesis pathway. OAZ1 in viruliferous insects is mostly recruited by RSMV M to facilitate viral assembly, which in turn competitively prevents the formation of OAZ1-ODC1 heterodimer and thus increases ODC1 homodimer, thereby promoting polyamine production to benefit viral infection. Thus, RSMV M, OAZ1 and ODC1 have formed a delicate regulatory circuit to co-ordinate polyamine biosynthesis and efficient viral propagation in insect vectors (Fig. 6). However, plant nucleorhabdovirus RYSV significantly inhibits OAZ1 expression in its leafhopper vector (Zhang et al., 2021)*,* suggesting that the assembly of RYSV particles in insect nuclei does not dependent on OAZ1.

Distantly-related rhabdovirus M proteins have similar structures and are presumed to form coiled helical structures to condense RNP cores, thereby conferring the rigidity of the non-enveloped virion structures (Gaudier et al., 2001; 2002; Graham et al., 2008). We observe that the N-terminus (50 aa) of RSMV M contains the conserved α-helices, which can self-associate to form the bacilliform structures. Though it is commonly proposed that the molecules of rhabdoviruses M proteins directly recognize and condense RNP cores to form the rigid bullet-shaped or bacilliform virions (Jayakar et al., 2004; Jackson et al., 2005), our results suggest that RSMV M itself may form the rigid bacilliform structures prior to directly packaging the RNP cores inside. We further determine that the interaction of the N-terminus of RSMV M with OAZ1 ensures the proper assembly of the rigid bacilliform structures by RSMV M. The rigid bacilliform virions with RSMV M on their surface are aligned in parallel and packed together to form the paracrystalline array via the filaments. Immunoelectron microscopy reveals that OAZ1 also is localized at the space between the adjacent bacilliform particles, and potentially it is one component of such filaments. Together, we deduce that OAZ1 would confer the ability for RSMV M to self-associate to assemble the bacilliform structures and then form aggregates. In addition to its interaction with ODC1 and an antizyme inhibitor, OAZ1 also targets and mediates the degradation or stabilization of various crucial regulatory proteins, including cyclinD1, Aurora-Akinase, Smad1, DeltaNp73, and Mps1 (Igarashi et al., 2015; Firpo & Mounce, 2020). Our finding that OAZ1 mediates the proper assembly of rigid bacilliform non-enveloped virions of a plant rhabdovirus is one new regulatory role of OAZ1. Apart from functioning as a structural component, rhabdovirus M proteins also play regulatory roles by controlling the switch between viral replication and transcription (Finke et al., 2003). It remains to be determined how the complex of cytorhabdovirus M-OAZ1 plays more regulatory roles during viral infection.

Many plant viruses with an obvious agricultural impact such as reoviruses, rhabdoviruses, tospoviruses and tenuiviruses, are transmitted by insect vectors in a persistent-propagative manner (Wang et al., 2020). Even in very well-studied mosquito systems, little is known about how viruses manipulate the cellular polyamine homeostasis to facilitate the persistent propagation of viruses in insect vectors. Polyamines are essential for normal cell growth and viability in eukaryotic cells (Bae et al., 2018; Handa et al., 2018). Viruses and insect vectors compete for intracellular polyamines (Mounce et al., 2017; Bae et al., 2018; Handa et al., 2018; Firpo & Mounce, 2020). For the first time, we find that virus could directly hijack the antizyme OAZ1 for viral assembly purpose in insect vectors; however, such process further increases ODC1 synthesis to promote polyamines production, avoiding to cause the obvious fitness costs to insect vectors. We speculate that such a unique model for benefiting rhabdovirus propagation by modulating polyamine homeostasis could be of more general importance for virus-insect interactions. Inhibition of the polyamine biosynthetic pathway of insect vectors may provide an important means of intervening in viral propagation and transmission.

## Materials and methods

### Insect, viruses, cells and antibodies

Nonviruliferous *R. dorsalis* individuals were collected from Guangdong Province, China and propagated at 25 ± 3 °C in an air-conditioned room. RSMV-infected rice plants were also collected from Guangdong Province, China and propagated via *R. dorsalis* transmission. Sf9 cells were cultured and maintained in growth medium. Polyclonal antibodies against M, N and G proteins of RSMV were prepared as previously described (Zhao et al., 2019). Polypeptides of OAZ1 (SHSTESRGVGKQEK) and ODC1 (YPGNRGSSIDKIAE) derived from *R. dorsalis* were conjugated to the carrier protein mariculture Keyhole Limpet Hemocyanin and injected into rabbits to generate the antibodies against OAZ1 and ODC1, respectively. These antibodies were produced by Genscript USA Innovation Company (Nanjing), which were approved by the Science Technology Department of Jiangsu Province, China with approval number SYXK (Su) 2018–0015.

### Yeast two-hybrid assay

Yeast two-hybrid assay was performed using the Matchmaker Gal4 Two-Hybrid System3 (Clontech) according to the manufacturer’s protocol. The full-length ORFs of RSMV-encoded proteins (N, P, P3, M, P6 and G), OAZ1 and ODC1 of *R. dorsalis*, the N-terminal segment (bp 1–382, OAZ1N) and C-terminal ODC-AZ domain (bp 383–643) of OAZ1, the full-length ORF of OAZ1 form Sf9 cells, as well as the M-N and M-C segments of RSMV M were amplified and constructed in the bait plasmid pGBKT7 or the prey plasmid pGADT7, respectively.

### GST pull-down assay

The full-length ORFs of RSMV M or OAZ1 of *R. dorsalis* were cloned into the pGEX-3x vector for expressing the GST fusion proteins (GST-M or GST-OAZ1). The full-length ORFs of OAZ1 or ODC1 of *R. dorsalis* were cloned into the pDEST17 vector for expressing the His fusion proteins (His-OAZ1 or His-ODC1). The recombinant proteins GST-M, GST-OAZ1, GST, His-OAZ1 and His-ODC1 were individually expressed in the *Escherichia coli* strain Rosetta. The GST-fused proteins were incubated with glutathione-Sepharose beads (Amersham) for 4 h at 4 °C, and the His-fused proteins were subsequently added to the beads and incubated for 2 h at 4 °C. After being centrifuged and washed, the bead-bound proteins were separated by SDS-PAGE and detected using antibodies against GST and His by western blot assays.

### Co-immunoprecipitation (co-IP)

Co-IP assay was performed using the Kit (Thermo Fisher Scientific) according to the manufacturer’s instructions. Briefly, the total proteins of 30 viruliferous or nonviruliferous *R. dorsalis* adults were lysed. RSMV-M-specific IgG was added to the Protein A affinity beads for immobilization for 2 h at room temperature. After being centrifuged and washed with phosphate buffer for three times, the lysates were subsequently added to the beads and incubated for 2 h at 4 °C. Then the nonbound proteins in the lysates were collected by centrifuging the Protein A affinity beads. The Protein A affinity beads were washed with phosphate buffer for three times and eluted with IgG elution buffer (0.1 M glycine, pH 2.8). The resultant immune complexes and nonbound proteins were separated by SDS-PAGE for western blot analysis using OAZ1- and β-actin-specific IgG.

### Polyamine biosynthesis assay

To analyze the effect of viral infection on polyamines production, 30 insects (nonviruliferous or viruliferous) were homogenized in 0.5 ml ice-cold 0.2 M perchloric acid, and then incubated for 30 min on ice. The extracts were centrifuged at 12, 000 rpm for 20 min, and then the supernatants were collected to detect the polyamine contents. The polyamines including PUT, SPD and SPM were analyzed by UPLC-MS/MS assay as previously described (Tsutsui et al., 2013). We then used a membrane-feeding approach for delivering DFMO to *R. dorsalis* to test whether DFMO treatment inhibited polyamines production and viral infection in insect vectors. Two hundred second-instar nymphs of *R. dorsalis* were fed on RSMV-infected rice plants for 2 days. Then 100 of these insects were fed with 10% sucrose and the remains were fed on 0.5 mM DFMO diluted in 10% sucrose, which were held within a double layer of parafilm in a plastic tube. After 4 days of feeding, insects were collected, and the concentrations of polyamines were analyzed by UPLC-MS/MS assay. The effect of DFMO treatment on the transcript levels of RSMV N was detected by RT-qPCR assay.

### Effects of OAZ1 on viral assembly and transmission by *R. dorsalis*

To track the expression of OAZ1 and ODC1 in RSMV-infected insects, 200 s-instar *R. dorsalis* individuals were fed on RSMV-infected rice plants for 2 days and then reared on healthy rice plants. At 4 days post-first access to diseased plants (padp), the internal organs of 30 insects (nonviruliferous or viruliferous) were dissected, fixed in 4% paraformaldehyde, immunolabeled with M-specific IgG conjugated to fluorescein isothiocyanate (FITC) (M-FITC), OAZ1-specific IgG conjugated to rhodamine (OAZ1-rhodamine), or ODC1-specific IgG conjugated to rhodamine (ODC1-rhodamine), and processed for immunofluorescence microscopy. At 4 days padp, the effects of RSMV infection on the transcript levels of OAZ1 and ODC1 genes in viruliferous or nonviruliferous insects were detected by RT-qPCR using the SYBR Green PCR Master Mix kit (Promega, USA). The actin transcript of *R. dorsalis* served as the internal reference and the relative gene expression levels were calculated using the 2^-ΔΔCT^ method (Livak & Schmittgen, 2001). At 4 days padp, we also detected the effects of RSMV infection on the protein levels of OAZ1 and ODC1 in viruliferous or nonviruliferous insects. In western blot assays, OAZ1-, ODC1-, M-, N- or β-actin-specific IgG (Sigma) served as the primary antibodies and goat anti-rabbit IgG-peroxidase served as the secondary antibodies.

To test the effects of knocking down OAZ1 expression by RNAi on the formation of bacilliform structures of RSMV M in *R. dorsalis,* the dsRNAs targeting a 643-bp region of the OAZ1 gene (dsOAZ1) were synthesized in vitro using the T7 RiboMAX Express RNAi System (Promega). To determine the function of OAZ1 on viral infection, 200 s instar individuals of *R. dorsalis* were microinjected with 0.5 μg/μl dsOAZ1 or dsRNAs targeting GFP gene (dsGFP) after feeding on RSMV-infected rice plants for 1 day, and then fed on healthy rice seedlings for 4 days. The survival rates of treated insects were recorded daily. The intestines from 30 insects were dissected, fixed, immunolabeled with M-FITC and OAZ1-rhodamine, and processed for immunofluorescence microscopy as previously described (Yu et al. 2021). The effects of dsRNAs on the transcript or protein levels of M and OAZ1 were detected by relative RT-qPCR or western blot assays.

### Baculovirus expression of RSMV M and OAZ1 in Sf9 cells

The baculovirus system was used to express OAZ1, RSMV M, and the N-terminal (bp 1–150, M-N) and C-terminal (bp 151–525, M-C) segments of RSMV M, as previously described (Jia et al., 2012). Recombinant bacmids containing OAZ1, RSMV M, M-N or M-C were transfected Sf9 cells in the presence of the Cellfectin II reagent (Thermo, USA). At 48 h post-infection (hpi), the Sf9 cells infected with recombinant bacmids were fixed in 4% paraformaldehyde and processed for immunofluorescence microscopy. The dsRNAs targeting a 537-bp segment of Sf-OAZ1 gene (dsSf-OAZ1) were synthesized in vitro (Fig. S9). Sf9 cells were transfected with 0.5 μg/μl dsRNAs targeting dsSf-OAZ1 or dsGFP in the presence of the Cellfectin II reagent for 6 h and then grew in fresh growth medium. Then, Sf9 cells were infected with recombinant baculovirus expressing RSMV M. At 48 hpi, the cells were fixed and processed for immunofluorescence microscopy to observe the effect of dsSf-OAZ1 on the distribution of RSMV M. In addition, the effect of dsSf-OAZ1 or dsGFP treatment on the transcript levels of Sf-OAZ1 gene were measured by RT-qPCR assays as described above.

### Viral transmission by *R. dorsalis*

To investigate the transmission rates of RSMV by *R. dorsalis* treated with dsOAZ1 or DFMO solution, more than 600 s instar individuals fed on RSMV-infected rice plants for 2 days. Then groups of 100 insects were each microinjected with dsOAZ1 or dsGFP, or fed with 10% sucrose or 0.5 mM DFMO diluted in 10% sucrose for 4 days. Subsequently, the insects fed on healthy rice seedlings. At 10 padp, the individual insects were transferred in glass tubes that each contained a single rice seedling for 2 days. Then 10 days later, the plants inoculated with viruliferous *R. dorsalis* were subjected to RT-PCR detection of RSMV N gene. The transmission rates of RSMV by *R. dorsalis* were calculated as the percentage of RT-PCR-positive plants out of the total number of plants.

### Immunoelectron microscopy

The intestines from *R. dorsalis* (viruliferous or nonviruliferous) or Sf9 cells infected with recombinant baculoviruses were fixed, dehydrated, embedded, and thin sections were cut as previously described (Wei et al., 2006). Sections were then incubated with N-, M-, OAZ1-, ODC1- or G-specific IgGs and immunolabeled with goat antibodies against rabbit IgG that had been conjugated with 15-nm-diameter gold particles (Sigma).

### Sequence alignment and phylogenetic analysis

Multiple sequence alignments were generated using MEGA5 and the phylogenetic trees for OAZ1 and ODC1 were generated using IQ-tree (Tamura et al., 2011; Nguyen et al., 2015). The amino acid sequences of the M proteins were submitted to the CUBIC PredictProtein server (http://cubic.bioc.columbia.edu/pp/) for secondary structure prediction.

### Statistical analyses

All data were analyzed with SPSS, version 17.0. Percentage data were arcsine square-root transformed before analysis as previously described (Yu et al. 2021). Multiple comparisons of the means were conducted based on Tukey’s honest significant difference (HSD) test using a two-way analysis of variance (ANOVA). Comparisons between two means were conducted using independent *t*-tests. The data were back-transformed after analysis for presentation in the text and figures.

## Supplementary Information

**Additional file 1: Fig. S1.** Sequence alignments of OAZ1 from *R. dorsalis* and other insect species. **A** A schematic diagram of the two ORFs of OAZ1 from *R. dorsalis*. A start codon (ATG), + 1 frameshift site (T) and a stop codon (TAG) are indicated. The ORF2 contains an ODC-AZ domain. **B** Comparison between the deduced amino acid sequences of OAZ1 from *R. dorsalis* and other insect species. Red arrow is the + 1 frameshift site. Red underline indicates the ODC-AZ domain site. **C** A phylogenetic tree of OAZ1 amino acid sequences from *R. dorsalis* and other insect species. The numbers at each branch indicate the percentage of times a node was supported in 1000 bootstrap replicates. The species names and GenBank accession numbers of the OAZ1 sequences are as follows: *Cimex lectularius* (XP_014240070.1), *Zootermopsis nevadensis* (KDR17620.1), *Drosophila melanogaster* (NP_725105.1), *Frankliniella occidentalis* (XP_026277816.1), *Recilia dorsalis* (MN931685), *Nephotettix cincticeps* (MN931686) and *Nilaparvata lugens* (XP_022185700.1). **Fig. S2.** Sequence alignments of ODC1 from *R. dorsalis* and other insect species. **A** Comparison between deduced amino acid sequences of ODC1 from *R. dorsalis* and other insect species. **B** A phylogenetic tree of ODC1 amino acid sequences from *R. dorsalis* and other insect species. The numbers at each branch indicate the percentage of times a node was supported in 1000 bootstrap replicates. The species names and GenBank accession number of the ODC1 sequences are as follows: *Recilia dorsalis* (MN854702), *Nephotettix cincticeps* (MN931687), *Nasonia vitripennis* (XP_001604390.2), *Laodelphax striatellus* (RZF47632.1) *Cephus cinctus* (XP_015586585.1), *Habropoda laboriosa* (XP_017793214.1), and *Drosophila melanogaster* (NP_477052.2). **Fig. S3.** Immunogold labeling of RSMV N, M or G in virus-infected intestines of *R. dorsalis*. Insect intestines were immunolabeled with N- (**A**), M- (**B, C**) or G- (**D**) specific antibodies and goat antibodies against rabbit IgG that had been conjugated with 15-nm-diameter gold particles as secondary antibodies. Red arrows mark gold particles. VM, viroplasm matrix; NV, non-enveloped virions; EV, enveloped virions. ER, endoplasmic reticulum. Bars, 100 nm. **Fig. S4.** Effects of RSMV infection on ODC1 expression in *R. dorsalis*. **A** Immunogold labeling of ODC1 in midguts of nonviruliferous and viruliferous insects. The midguts were immunolabeled with ODC1-specific IgG and goat antibodies against rabbit IgG that had been conjugated with 15-nm-diameter gold particles (arrows) as secondary antibodies. Bars, 100 nm. **B** At 4 days padp, the midguts of nonviruliferous or viruliferous insects were immunolabeled with M-FITC (green) and ODC1-rhodamine (red) and then examined by immunofluorescence microscopy. NV, non-enveloped virions; fc, filter chamber; amg, anterior midgut. Bars, 30 μm. **Fig. S5.** The effect of the knockdown of OAZ1 by RNAi on the mortality rates of *R. dorsalis*. The mortality rates of nonviruliferous *R. dorsalis* individuals after treatment with dsGFP or dsOAZ1 were analyzed at different days. Each histogram bar represents the mean (± standard deviation) from three independent replicates. **Fig. S6.** The amino acid sequence of the RSMV M was submitted to the CUBIC PredictProtein server (http://cubic.bioc.columbia.edu/pp/) for secondary structure prediction. The α-helix and β-strand are labeled with red columns and yellow arrows below the sequence, respectively. Predicted phosphorylation sites are labeled by green boxes. The hydrophobic residues Leu (L), IIe (I) and Val (V) are boxed. The Cys (C) residues are labeled in yellow. **Fig. S7.** Comparison between amino acid sequences of OAZ1 from *R. dorsalis* and *S. frugiperda*. Amino acid sequences of OAZ1 from *S. frugiperda* shared 39.82% similarity with that from *R. dorsalis*.

**Additional file 2: Table S1.** Transmission rates of RSMV by insect vectors that were treated with DFMO or dsOAZ1.

## Data Availability

The data and materials that support the findings of this study are available from the corresponding author upon request.
