# Supplementary material for: Polyamine-metabolizing enzymes are activated to promote the proper assembly of rice stripe mosaic virus in insect vectors
Source: Stress Biol. 2022 Apr 15;2(1):10. doi: 10.1007/s44154-021-00032-z (PMC10441986; doi:10.1007/s44154-021-00032-z)
Supplement: Supplementary file 1 — Additional file 1: Fig. S1. Sequence alignments of OAZ1 from R. dorsalis and other insect species. A A schematic diagram of the two ORFs of OAZ1 from R. dorsalis. A start codon (ATG), + 1 frameshift site (T) and a stop codon (TAG) are indicated. The ORF2 contains an ODC-AZ domain. B Comparison between the deduced amino acid sequences of OAZ1 from R. dorsalis and other insect species. Red arrow is the + 1 frameshift site. Red underline indicates the ODC-AZ domain site. C A phylogenetic tree of OAZ1 amino acid sequences from R. dorsalis and other insect species. The numbers at each branch indicate the percentage of times a node was supported in 1000 bootstrap replicates. The species names and GenBank accession numbers of the OAZ1 sequences are as follows: Cimex lectularius (XP_014240070.1), Zootermopsis nevadensis (KDR17620.1), Drosophila melanogaster (NP_725105.1), Frankliniella occidentalis (XP_026277816.1), Recilia dorsalis (MN931685), Nephotettix cincticeps (MN931686) and Nilaparvata lugens (XP_022185700.1). Fig. S2. Sequence alignments of ODC1 from R. dorsalis and other insect species. A Comparison between deduced amino acid sequences of ODC1 from R. dorsalis and other insect species. B A phylogenetic tree of ODC1 amino acid sequences from R. dorsalis and other insect species. The numbers at each branch indicate the percentage of times a node was supported in 1000 bootstrap replicates. The species names and GenBank accession number of the ODC1 sequences are as follows: Recilia dorsalis (MN854702), Nephotettix cincticeps (MN931687), Nasonia vitripennis (XP_001604390.2), Laodelphax striatellus (RZF47632.1) Cephus cinctus (XP_015586585.1), Habropoda laboriosa (XP_017793214.1), and Drosophila melanogaster (NP_477052.2). Fig. S3. Immunogold labeling of RSMV N, M or G in virus-infected intestines of R. dorsalis. Insect intestines were immunolabeled with N- (A), M- (B, C) or G- (D) specific antibodies and goat antibodies against rabbit IgG that had been conjugated with [file 44154_2021_32_MOESM1_ESM.pdf]

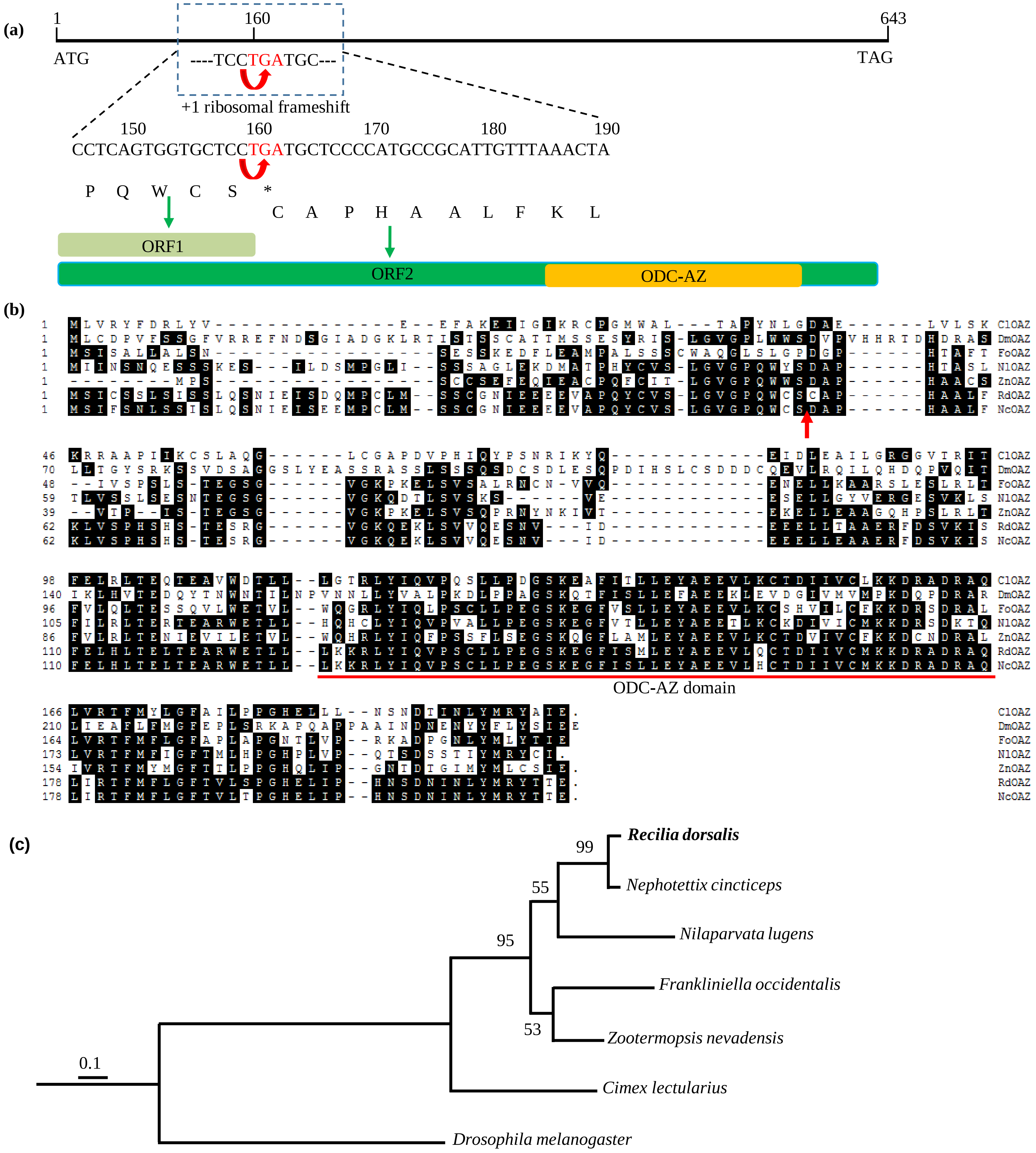
(a)
1
160
643
ATG
----TCCTGATGC---
TAG
+1 ribosomal frameshift
150
160
170
180
190
CCTCAGTGGTGCTCCTGATGCTCCCCATGCCGCATTGTTTAAACTA
P Q W C S *
C A P H A A L F K L
ORF1
ORF2
ODC-AZ
(b)
ClOAZ
DmOAZ
FoOAZ
NlOAZ
ZnOAZ
RdOAZ
NcOAZ
ODC-AZ domain
(c)
Recilia dorsalis
Nephotettix cincticeps
Nilaparvata lugens
Frankliniella occidentalis
Zootermopsis nevadensis
Cimex lectularius
Drosophila melanogaster
99
55
95
53
0.1

Figure S1

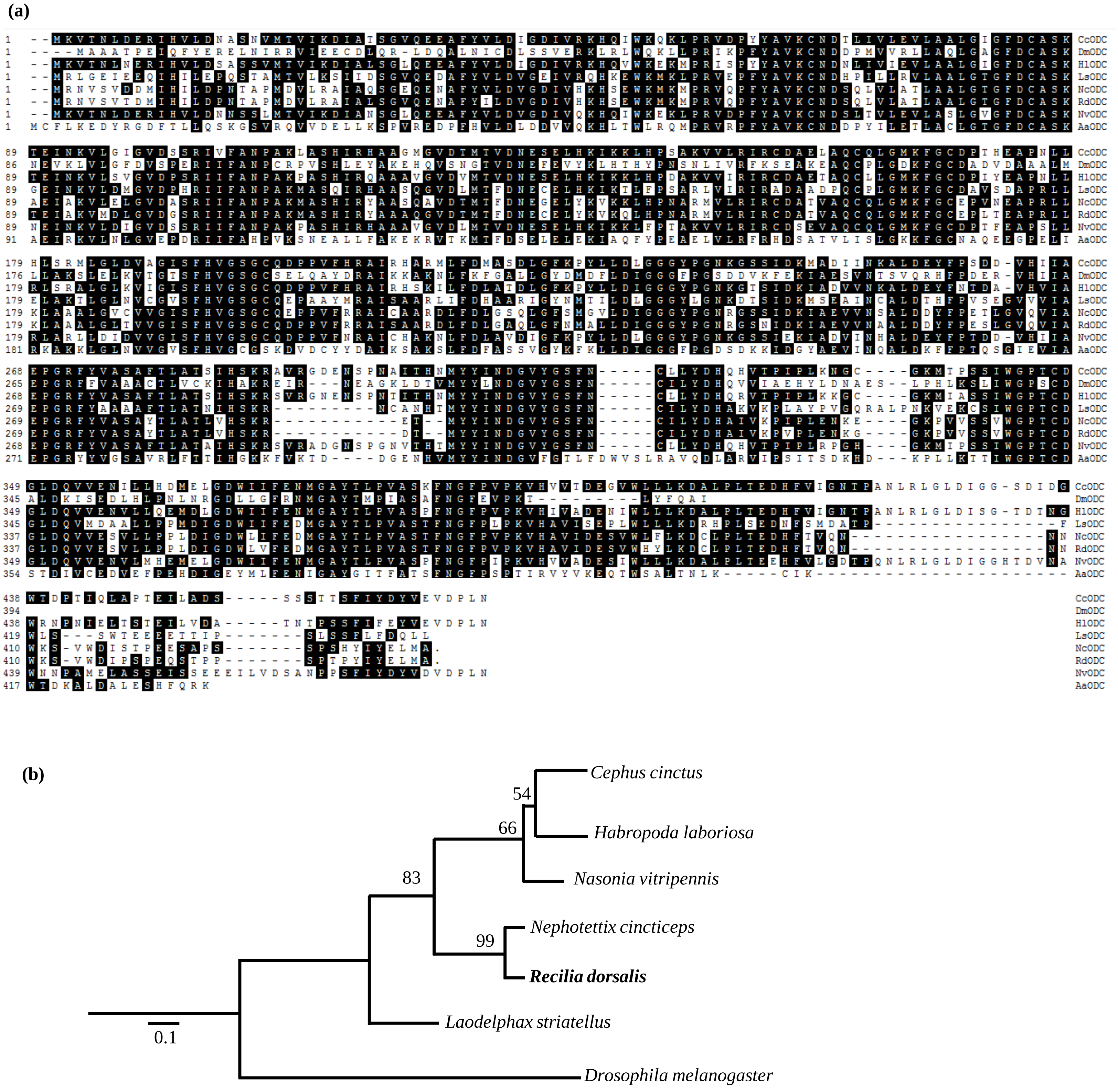
(a)
CcODC
DmODC
HlODC
LsODC
NcODC
RdODC
NvODC
AaODC
(b)
Cephus cinctus
Habropoda laboriosa
Nasonia vitripennis
Nephotettix cincticeps
Recilia dorsalis
Laodelphax striatellus
Drosophila melanogaster
54
66
83
99
0.1

Figure S2

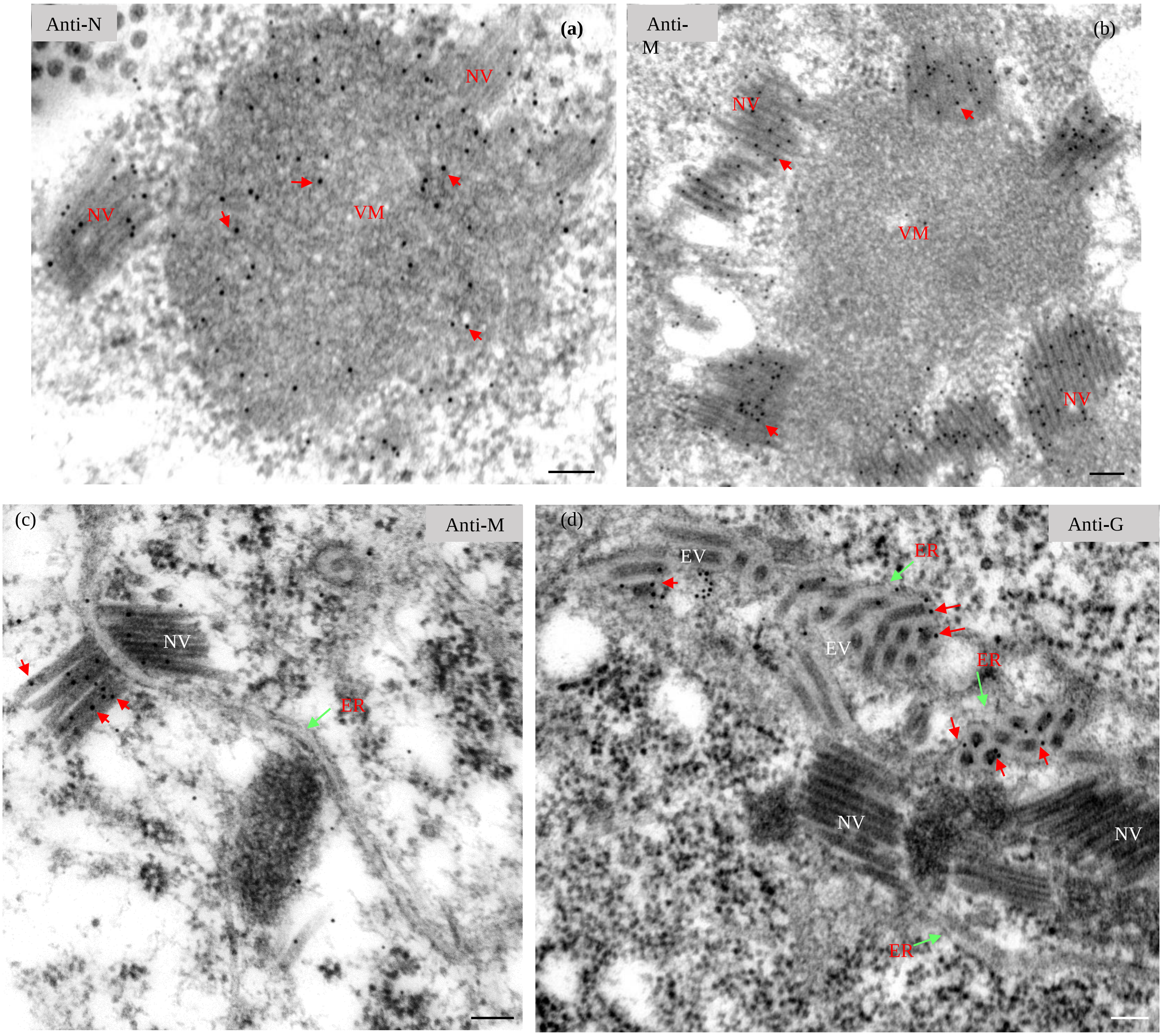
Anti-N
(a)
NV
NV
VM
Anti-
M
(b)
NV
VM
NV
(c)
Anti-M
NV
ER
(d)
Anti-G
EV
ER
EV
ER
NV
NV
ER

Figure S3

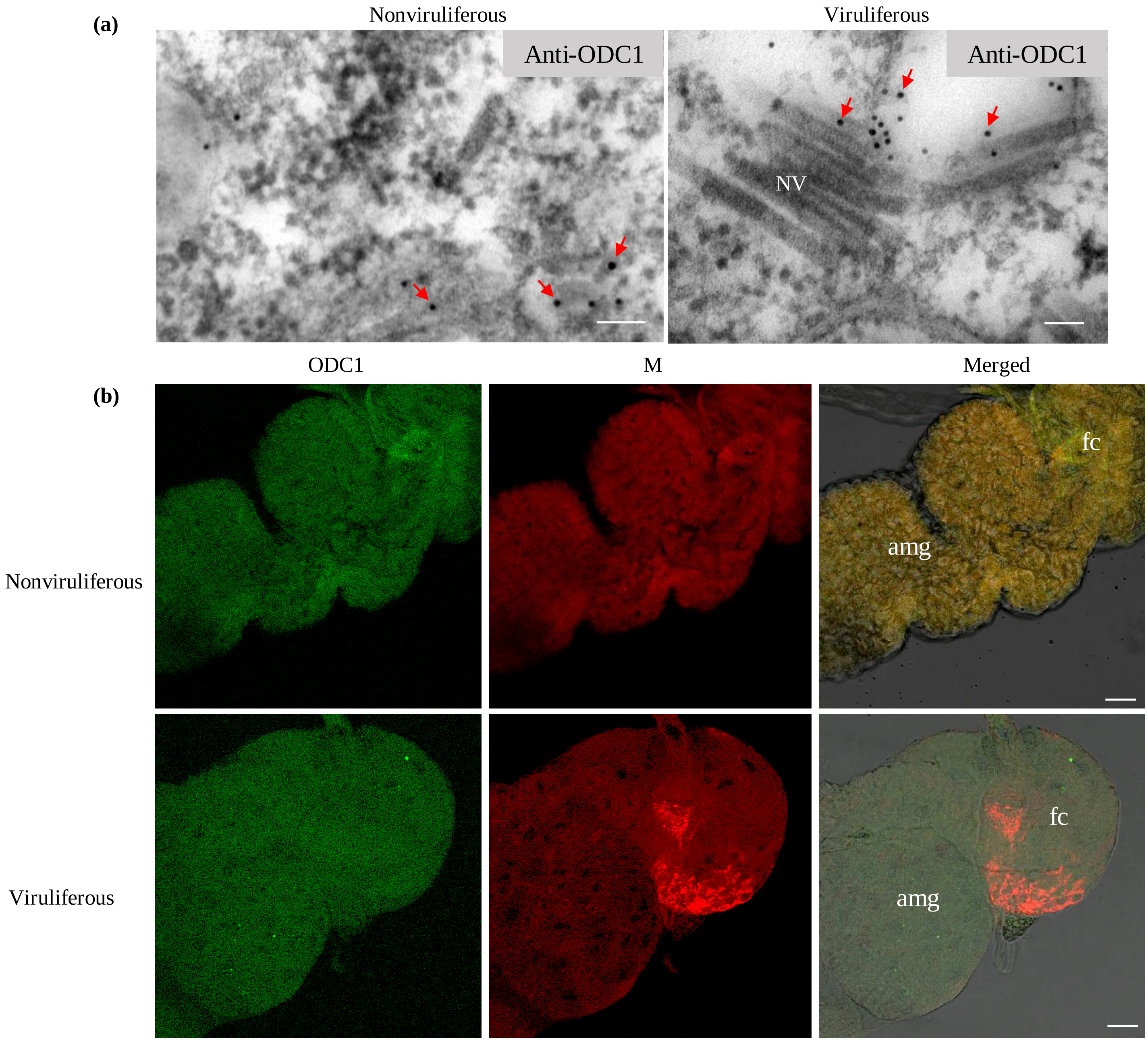
(a)
Nonviruliferous
Viruliferous
Anti-ODC1
Anti-ODC1
NV
ODC1
M
Merged
(b)
Nonviruliferous
Viruliferous
fc
amg
fc
amg

Figure S4

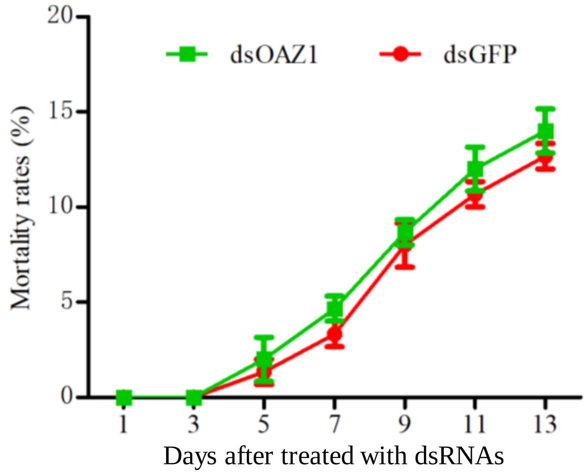
20
15
10
5
0
Mortality rates (%)
dsOAZ1
dsGFP
1
3
5
7
9
11
13
Days after treated with dsRNAs

Figure S5

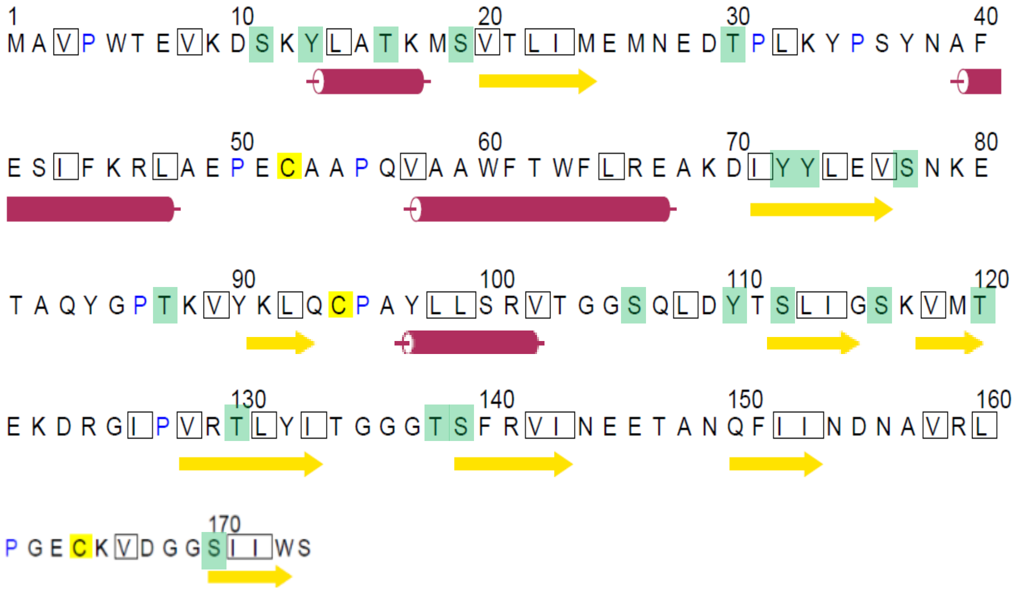

Figure S6

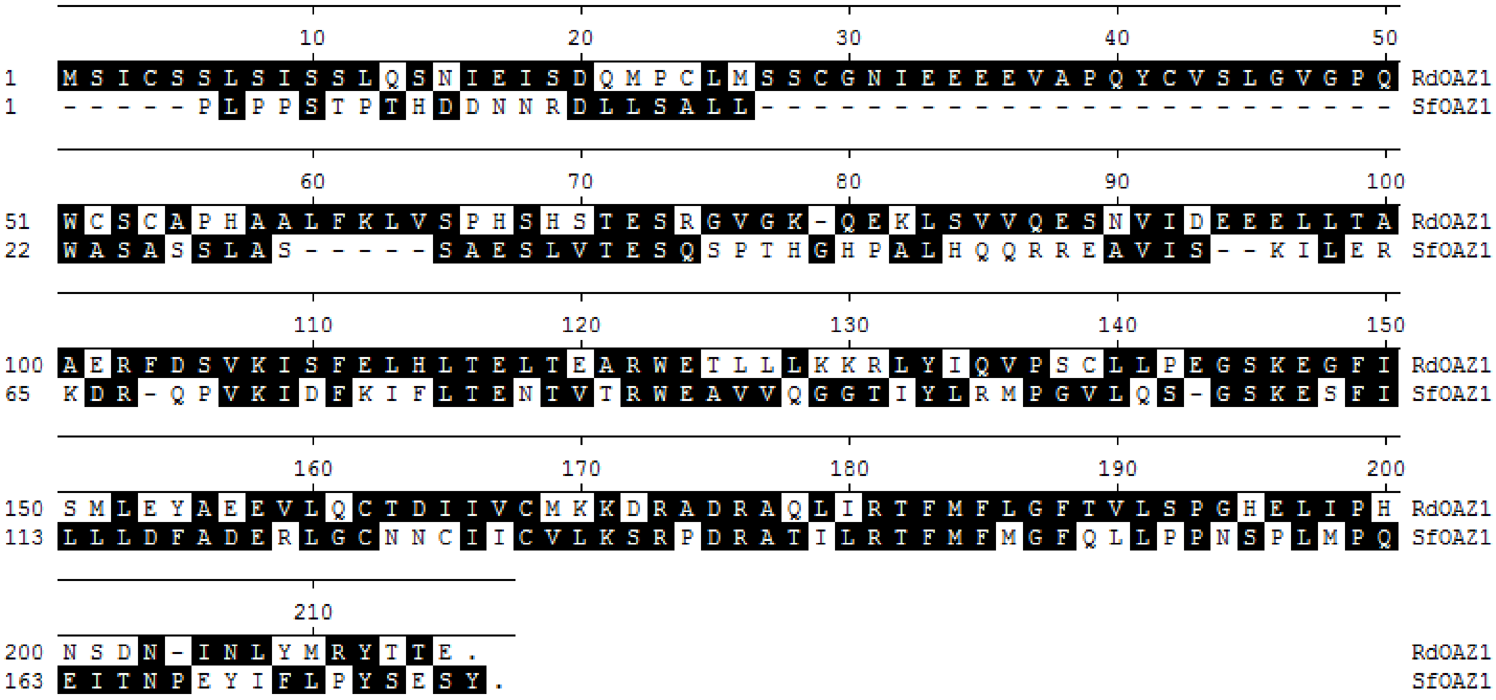
1 MSICSSLSISSLQSNIEISDQMPCLMSSCGNIEEEEVAPQYCVSLGVGPQ RdOAZ1
1 -----PLPPSTPTHDDNNRDLLSALL------------------------ SfOAZ1
51 WCSCAPHAALFKLVSPHSHSTESRGVGK-QEKLSVVQESNVIDEEELLTA RdOAZ1
22 WASASSLAS-----SAESLVTESQSPTHGHPALHQQRREAVIS--KILER SfOAZ1
100 AERFDSVKISFELHLTELTEARWETLLLKKRLYIQVPSCLLPEGSKEGFI RdOAZ1
65 KDR-QPVKIDFKIFLTENTVTRWEAVVQGGTIYLRMPGVLQS-GSKESFI SfOAZ1
150 SMLEYAEEVLQCTDIIVCMKKDRADRAQLIRTFMFLGFTVLSPGHELIPH RdOAZ1
113 LLLDFADERLGCNNCIICVLKSRPDRATILRTFMFMGFQLLPPNSPLMPQ SfOAZ1
200 NSDN-INLYMRYTTE. RdOAZ1
163 EITNPEYIFLPYSESY. SfOAZ1

Figure S7
