# Supplementary material for: Polyamine-metabolizing enzymes are activated to promote the proper assembly of rice stripe mosaic virus in insect vectors
Source: Stress Biol. 2022 Apr 15;2(1):10. doi: 10.1007/s44154-021-00032-z (PMC10441986; doi:10.1007/s44154-021-00032-z)
Supplement: Supplementary file 2 — Additional file 2: Table S1. Transmission rates of RSMV by insect vectors that were treated with DFMO or dsOAZ1. [file 44154_2021_32_MOESM2_ESM.doc]

Supplementary Table 1. Transmission rates of RSMV by insect vectors that were treated with DFMO or dsOAZ1.

| Treatments | No. of viruliferous insects (n=30) | | |  | No. of viruliferous plants (n=100) | | | Transmission rates (%) |
| --- | --- | --- | --- | --- | --- | --- | --- | --- |
|  | I | II | III |  | I | II | III |  |
| DFMO | 10 | 12 | 11 |  | 20 | 25 | 27 | 24 |
| Sucrose | 11 | 13 | 14 |  | 48 | 55 | 53 | 52 |
| dsOAZ1 | 8 | 6 | 9 |  | 3 | 2 | 4 | 3 |
| dsGFP | 12 | 15 | 13 |  | 46 | 53 | 51 | 50 |
